# Adult-Onset Case of Undiagnosed Neurodegeneration with Brain Iron Accumulation with Psychotic Symptoms

**DOI:** 10.1155/2014/742042

**Published:** 2014-05-20

**Authors:** Luigi Attademo, Enrico Paolini, Francesco Bernardini, Roberto Quartesan, Patrizia Moretti

**Affiliations:** ^1^School of Psychiatry, University of Perugia, 06156 Perugia, Italy; ^2^Division of Psychiatry, Clinical Psychology and Psychiatric Rehabilitation, Department of Clinical and Experimental Medicine, Santa Maria della Misericordia Hospital, University of Perugia, 06156 Perugia, Italy

## Abstract

Neurodegeneration with brain iron accumulation (NBIA) is a collective term to indicate a group of neurodegenerative diseases presenting accumulation of iron in the basal ganglia. These disorders can result in progressive dystonia, spasticity, parkinsonism, neuropsychiatric abnormalities, and optic atrophy or retinal degeneration. Onset age ranges from infancy to late adulthood and the rate of progression is very variable. So far, the genetic bases of nine types of NBIA have been identified, pantothenate-kinase-associated neurodegeneration (PKAN) being the most frequent type. The brain MRI “eye-of-the-tiger” sign, T2-weighted hypointense signal in the *globus pallidus* with a central region of hyperintensity, has been considered virtually pathognomonic for PKAN but recently several reports have denied this. A significant percentage of individuals with clinical and radiographic evidence of NBIA do not have an alternate diagnosis or mutation of one of the nine known NBIA-associated genes (idiopathic NBIA). Here we present an adult-onset case of “undiagnosed” NBIA with the brain MRI “eye-of-the-tiger” sign, and with psychotic symptoms which were successfully treated with antipsychotic and mood stabilizer medications. Here, the term “undiagnosed” is used because the patient has not been screened for all known NBIA genes, but only for two of them.

## 1. Introduction

Neurodegeneration with brain iron accumulation (NBIA) is a collective term to indicate a group of neurodegenerative diseases characterized by abnormal accumulation of iron in the basal ganglia (most often in the* globus pallidus* and/or* substantia nigra*) and progressive extrapyramidal clinical manifestations [1].

The most common form of NBIA seems to be pantothenate-kinase-associated neurodegeneration (PKAN), accounting for 35–50% of NBIA cases [2]. The worldwide prevalence of PKAN has been estimated at a ratio of 1 : 1.000.000 [3], with no race or gender related variations [4].

PKAN can be classified either as classic (typical) PKAN or as late-onset (atypical) PKAN [3, 5]. The typical form of the disease, with onset in the first decade of life, is characterized by rapidly progressing gait impairment, focal dystonia, pyramidal dysfunction, pigmentary retinopathy, and cognitive impairment and with loss of ambulation occurring within 10–15 years from the onset. The atypical form, with onset in the second or third decade, is characterized by psychiatric symptoms and focal dystonia (with or without parkinsonism or chorea, cognitive impairment, and late gait dysfunction), as well as by a slower progression and eventually by loss of ambulation occurring from 15 to 40 years after the onset [5]. Both forms of PKAN share a sign in brain MRIs: the “eye-of-the-tiger” sign, a T2-weighted hypointense signal in the* globus pallidus* with a central region of hyperintensity [1]. Although 15% of cases are sporadic [4], PKAN is an autosomal recessive disorder caused by mutations in the PANK2 gene located in chromosome 20p13 [3].

As well as PKAN, other conditions are presently considered as part of the NBIA group of disorders: PLA2G6-associated neurodegeneration (PLAN), neuroferritinopathy, aceruloplasminemia, FAHN, MPAN, BPAN, Kufor-Rakeb, and other more rare disorders with an identified genetic background [1]. Instead, individuals with clinical, radiographic, and sometimes pathologic evidence of NBIA, but without a mutation of one of the nine known NBIA-associated genes, should be classified as idiopathic NBIA [1].

Idiopathic NBIA is of unknown origin, although it is suspected to be genetic. In many families, the person diagnosed with NBIA is the first and only affected individual. The symptoms are more varied because there are probably several different causes for the neurodegeneration in the individuals of this group. As with other forms of NBIA, the idiopathic form features early- and late-onset types.

As previously said, there is a specific pattern in brain MRIs, called the “eye-of-the-tiger” sign which, in the past, was considered to be virtually pathognomonic for PKAN [6]. This sign used to be considered as having a one-to-one correlation with the presence of a positive PANK2 gene mutation; however, more recently, several PANK2 negative “eye-of-the-tiger” cases have also been reported [5, 7, 8]. Most of the PANK2 negative “eye-of-the-tiger” sign cases were in fact late-onset or atypical ones [5, 7].

Although psychiatric symptoms (e.g., cognitive deficit, personality changes with impulsivity and violent outbursts, depression, anxiety, emotional lability, and obsessive compulsive disorder) are common in the atypical form [2, 5], psychosis has rarely been reported as a major symptom of PKAN [4, 9]. Here, we present an interesting case of “undiagnosed” NBIA. This case was genetically PANK2 (and PLA2G6) negative, but it showed the “eye-of-the-tiger” sign in the MRI and the clinical features of an atypical form of PKAN. The patient also showed psychotic symptoms which improved after treatment with antipsychotic and anticonvulsant/mood stabilizer medications.

## 2. Case Presentation

Our patient is a Caucasian 48-year-old woman, who was voluntarily admitted to the Psychiatric Diagnosis and Care Service (inpatients unit), in a condition of manic state and pathological behaviour.

The patient had no family history of psychiatric disorders. Her psychophysical development was normal; after compulsory schooling, she carried on to obtain a high school diploma. By the age of 18, she had been diagnosed for a Schizoaffective disorder and treated in a community mental health outpatients service. In the following years, she was admitted several times in psychiatric inpatients unit. For many years, she was treated with first generation antipsychotics (in particular haloperidol and chlorpromazine). During her youth, the patient presented a periodic substance abuse (heroin, cannabis, and alcohol). She was indeed also treated with methadone antiaddictive therapy. Moreover, during her youth, the patient was reported for an oculogyration of uncertain origin (probably antipsychotics-related), as well as for cervical dystonia and dystonia in flexion of the left* hallucis*, which appeared at the age of 35 after therapy with high doses of first generation antipsychotics (haloperidol and chlorpromazine). These conditions were treated with botulinum toxin first and by a movement disorder unit for the followup. At the age of 42, she was diagnosed with a brain and cervical rachis MRI showing wide-spread white matter hyperintensity in both cerebral hemispheres. Two years later, a new brain and* sella turcica* MRI showed a focal area of hypointensity in the central area of the anterior pituitary and a bilateral signal distortion on the* globus pallidus*, which presented hyperintensity in the central area and high hypointensity on the edge, with the typical “eye-of-the-tiger” sign and concomitant bilateral hypointensity of the* substantia nigra*. Moreover, a widely extended mild cortico-subcortical atrophy was also detected. Because of the evidence shown by the brain MRI, NBIA was suspected, hence the suggestion for a genetic test. The latter (search for gene mutation in PANK2 and PLA2G6) resulted negative, also ruling out possible duplications and/or deletions of whole exon in heterozygosity.

Before the admission to our acute inpatients unit, the patient was living with a friend who shared an apartment with her; she did not work and she received a disability subsidy. Earlier, the patient, who after her mother passed away lived for many years with her aunt, was living alone. As reported by her aunt, her cognitive level and ability in daily living activities were not significantly impaired before being admitted to our unit.

In the week prior to the admission to our acute inpatients unit, the patient presented with insomnia, irritability, logorrhoea, increased energy, and mental confusion. In the months before hospitalization, the patient had irregularly taken the psychopharmacological therapy (risperidone 6 mg/qd and delorazepam 4 mg/qd). At the moment of admission, she was alert, oriented in time and space. Her mood was euphoric, with incoherent and pressured speech. She also showed auditory hallucinations and delusions, loosening of associations, tangential thinking, and derailment of thought. On presentation, the neurological examination revealed a moderate dystonia in flexion of the left* hallucis*, and a mild tremor was present on the upper extremities; reflexes were conserved and she was able to ambulate independently. Verbal and motor stereotypies were also detected as well as bizarre gestures and behaviours, short-term memory disturbances, and concentration deficiencies. Ophthalmologic examination showed no alterations. The ECG resulted normal. Haematobiochemical exams resulted within the norm (including complete blood count, liver enzymes, renal function, glycemia, electrolytes, fat levels, ferritin, ceruloplasmin, oestrogens, progesterone, thyroid hormones, EMA IgA, and anti-tTG IgA) with the exception of monocytes (10.1%), ammonium (16 *μ*g/dL), total proteins (6.0 *μ*g/dL), prolactin (118.0 ng/mL), and a high anti-HCV antibody titer. No traces of psychotropic drugs were found in the urine samples. A risperidone 6 mg/qd and diazepam 10 mg/qd therapy was prescribed, which was later integrated with chlorpromazine 150 mg/qd, valproic acid 1000 mg/qd, and orphenadrine 100 mg/qd. After ten days, the patient showed signs of visible improvement. Her mood resulted fairly stable and her speech was fluid, less accelerated, and verbose. There were no signs of oddities, misperceptions, and alterations in the form and content of her thought. There was an overall motor improvement with a visible progressive reduction of dystonia and motor stereotypies. After about three weeks of hospitalization, the patient was discharged and returned to the care of the mental health outpatients service. During her hospitalization, the patient underwent a new brain MRI, which confirmed the signal alteration in both* globi pallidi* showing in the T2 sequences hyperintensity in the central portion and net hypointensity in the periphery, with the typical “eye-of-the-tiger” aspect. Moreover, in the same sequences, hypointensity was detected bilaterally in the* substantia nigra*. The aforementioned reports were radiologically compatible with NBIA.

After the discharge, the patient went back to live with a friend and, presently, she is going on with the prescribed therapy, without showing new psychotic symptoms or showing mood dyscontrol and without reported movement disturbances. No new hospitalizations were needed at present day.

## 3. Discussion

Our interpretation of the patient's condition is of a psychotic symptomatology within the context of an “undiagnosed” form of NBIA presenting the clinical characteristics of an atypical PKAN. The absence of currently identifiable genetic mutations in genes PANK2 and PLA2G6 allowed excluding PKAN and PLAN. The negative family case history ruled out the hypothesis of a neuroferritinopathy that is transmitted in an autosomal dominant manner.

Clinical exams and neuroimaging techniques performed on the patient allowed excluding other forms of NBIA, such as FAHN and aceruloplasminemia (without, however, carrying out the genetic investigations for these conditions). The related manifestation, the presence of focal dystonias and of psychiatric disturbances, the slow progression of the pathology as deduced from the anamnesis, and the “eye-of-the-tiger” sign in the MRI—without balance-related problems, pyramidal dysfunction, pigmentary retinopathy, and severe cognitive deterioration—allowed us to determine a diagnosis of “undiagnosed” NBIA with atypical features. Certainly, a great limitation of our case report is that the patient has not been screened for all known NBIA genes.

The main psychiatric symptoms found in the present case study are the typical ones of psychotic conditions such as disorganized speech, verbal and motor stereotypies and affectation, behavioural oddities, auditory hallucinations, and delusions with alterations in the cognitive sphere (disorientation and short-term memory delay) and in the emotional sphere (euphoria and logorrhoea).

It has been widely proved that basal ganglia are involved in the outset of the psychiatric manifestations of schizophrenia. In consideration of the fact that from a pathogenetic standpoint NBIA is characterized by an abnormal accumulation of iron at cerebral level, with a greater prevalence in the* globus pallidus* and in the* substantia nigra*, it can be inferred that the psychotic symptoms, mood disorders, cognitive deficits, and motor alterations are probably caused by the lesions in this area [9]. However, another limitation of this report is that the diagnostic procedure did not include a MRI-spectroscopy, looking for the presence of iron in the hypointense pallidal regions.

In this case study, a combination of typical and atypical antipsychotics allowed resolving the productive psychotic symptomatology with partial improvement in the cognitive sphere. By introducing an anticonvulsant/mood stabilizing agent, the alterations in the emotional sphere were also improved. Also neurological movement disorders, such as focal dystonia, upper extremities' tremor, and motor stereotypies, improved but the introduction of an anticholinergic/spasmolytic drug was necessary.
